# Antimicrobial and wound healing potential of naphthoquinones encapsulated in nanochitosan

**DOI:** 10.3389/fbioe.2023.1284630

**Published:** 2024-01-04

**Authors:** Cyntia Silva Freitas, Patricia Ribeiro Pereira, Raiane Vieira Cardoso, Fernanda Petzold Pauli, Ruan Carlos Busquet Ribeiro, Fernando De Carvalho Da Silva, Vitor Francisco Ferreira, Vania Margaret Flosi Paschoalin

**Affiliations:** ^1^ Advanced Analysis Laboratory in Biochemistry and Molecular Biology, Department of Biochemistry, Chemistry Institute, Federal University of Rio De Janeiro, Programa de Pós-Graduação em Ciência de Alimentos, Rio de Janeiro, Brazil; ^2^ Department of Biochemistry, Chemistry Institute, Federal University of Rio De Janeiro, Programa de Pós-Graduação em Química, Rio de Janeiro, Brazil; ^3^ Applied Organic Synthesis Laboratory, Department of Organic Chemistry, Chemistry Institute, Federal Fluminense University, Niterói, Brazil; ^4^ Department of Pharmaceutical Technology, Faculty of Pharmacy, Federal Fluminense University, Niterói, Brazil

**Keywords:** 3-chloromethylene-menadione, 2,3-dichloro-1,4-naphthoquinone, *Staphylococcus spp*, *Pseudomonas aeruginosa*, *Streptococcus pyogenes*, therapeutic index, human fibroblast cells susceptibility, nanocapsules

## Abstract

**Introduction:** The use of chitosan in pharmaceutical formulations is an advantageous approach due to this compound intrinsic biodegradability and biocompatibility, as well as ready availability and low polymer cost.

**Methods:** Herein, the naphthoquinones 3- chloromethylene-menadione (NQ1) and 2,3-dichloro-1,4-naphthoquinone (NQ2) were nanoencapsulated into chitosan (CNP) by the ionotropic gelatinization technique and characterized by DLS, FTIR, SEM, TGA and DSC, and their release profiles evaluated. The antimicrobial and wound healing activities were investigated.

**Results and Discussion:** Homogeneous chitosan nanocapsulses of about 193 nm and Z potential ranging from +30.6 to +33.1 mV loaded with NQ1 (CNP-NQ1) or NQ2 (CNPQNQ2). With nanoencapsulation efficiencies of ≥ 96%, the solubility of naphthoquinones in aqueous environments was improved, making them suitable for biological system applications. The encapsulated naphthoquinones displayed a controlled release of approximately 80% for CNP-NQ1 and 90% for CNP-NQ2 over an 8 h period at 36°C. Both CNP-NQ1 and CNP-NQ2 retained the already established free naphthoquinone antimicrobial activity against two *Staphylococcus aureus* strains, *Staphylococcus epidermidis*, *Streptococcus pyogenes* and *Pseudomonas aeruginosa*. Although presenting low toxicity to healthy human cells, only CNP-NQ1 displayed therapeutic indices above 100 for *S. aureus* and *S. epidermidis* and above 27 for *S. pyogenes* and *P. aeruginosa*, allowing for safe use in human tissues. Furthermore, CNP-NQ1 did not impair the migration of human fibroblast cells in scratch assays, adding promising wound healing properties to this formulation. These findings emphasize that CNP-NQ1 may be useful in protecting injured skin tissue from bacterial contamination, avoiding skin infections not only by reducing bacterial loads but also by accelerating the healing process until complete dermal tissue recovery.

## 1 Introduction

Nature comprises the primary reservoir of known organic compounds, with plants specifically emerging as a prominent and vital source of active molecules. Many natural plant substances are used as food preservatives and as templates for the development of modern synthetic drugs (Rahmoun et al., 2013; Zakir and Freitas, 2015). Quinones, in particular, have been investigated and reported for decades as pharmacological drugs, considered essential medicinal compounds by the World Health Organization (WHO) due to their various biological activities (Organization, 2020).

Quinones are widely distributed not only in plants, but also in animals and microorganisms, where they play vital roles in the biochemistry of energy production, due to their role as linkage compounds between electron transport carriers in the aerobic respiratory chain of living cells (Silva et al., 2020). These compounds are distinguished by their aromatic ring featuring two ketone substitutions. Both natural quinones and their analogues present cytotoxicity against microorganisms, due to their capacity to generate and donate free radicals. They also display the capability to form irreversible complexes with amino acids within protein chains, resulting in their inactivation. These distinctive properties empower quinones to target surface adhesions, such as cell wall polypeptides, as well as membrane enzymes, while also sequestering essential substrates necessary for microorganism survival (Othman et al., 2019; Silva et al., 2020). The pharmacological importance of this class of substances is verified in several drugs containing the quinoid nucleus in their structures, such as anthracycline antibiotics and several antitumoral drugs, including daunorubicin, doxorubicin, idarubicin and epirubicin (Monks and Jones, 2002; McGowan et al., 2017).

The most important and widely distributed chemical quinone is 1,4-naphthoquinone, and its derivatives exhibit different pharmacological effects, including anti-allergic (Lien et al., 2002), antifungal (Inbaraj and Chignell, 2004), anti-inflammatory (Sasaki et al., 2002), antithrombotic (Jin et al., 2004), antiplatelet (Lien et al., 2002), antiviral (Inbaraj and Chignell, 2004) properties, as well as antimicrobial (Inbaraj and Chignell, 2004) and antitumoral activities, stimulating enormous research interest in this compound class (Wellington, 2015; Pereyra et al., 2019). The biological activities and structural properties of 1,4-naphthoquinone compounds make these structures extremely useful in medicinal chemistry. Their low aqueous solubility, however, prevents them from being applied topically through conventional formulations, and therapeutic applications require attention due to their toxicity to biological systems (Silva et al., 2020). A novel approach to circumvent these disadvantages is the development of nanostructured therapeutic formulations employing biocompatible and biodegradable nanocapsules able to carry and deliver quinones to the bloodstream and intracellular aqueous environments (Ali and Ahmed, 2018).

Polymeric particles built in nanometric dimensions have been adopted to carry pharmaceuticals across extracellular and intracellular membranes due to their nanometric dimensions and controlled and targeted release ability. Chitosan, particularly in the form of its nanocounterparts, has been successfully applied in several therapeutic drug formulations. This compound is synthesized from natural chitin by partial N-deacetylation, and its low cytotoxicity, safety and biocompatibility towards human tissues and cells have been demonstrated by many *in vitro* and *in vivo* assays (Reis et al., 2006; Aguila et al., 2012; Dilbaghi et al., 2013; Gomes et al., 2014; Aluani et al., 2017; Badano et al., 2019; Bamburowicz-Klimkowska et al., 2019; Elshaarawy et al., 2019; Ahmed et al., 2020; Ferreira et al., 2022). Chitosan exhibits several inherent pharmacological activities that can be enhanced and amplified by chemical modifications or physicochemical interactions, contributing to chitosan applications in health promotion, particularly benefiting and enhancing skin tissue repair and regeneration. Chitosan has been used to prepare nanomaterials with mucoadhesive properties, as its positive charges allow for interactions with negative mucin charges, resulting in better mucosal tissues and epithelial cells contact. Furthermore, the positive charge of this polymer can promote paracellular transport by regulating tight-junctions (Felt et al., 1998; Onoue et al., 2014; Gomes et al., 2018). Chitosan-based material affects all wound healing phases, including bleeding control and inflammatory cell stimulation, accelerating these processes and culminating in the production of chitosan-based dressings. The healing effects of chitosan-based dressings may be modulated to accelerate hemostasis or other steps, depending on the dressing structure, *i.e.,* films, sponges, hydrogels, hydrocolloids, membranes, fibers, scaffolds and nanoparticles, also combined with different functional materials, such as gelatin, alginate, polyvinyl alcohol (PVA), carboxymethylchitosan (CMCS), cellulose or bioactive molecules (Patrulea et al., 2015; Hu et al., 2018; Muchová et al., 2021). Various commercial chitosan-based formulations are currently in use for wound treatments, such as replaceable dressings that focus on one function, for example, Hemcon^®^ Bandage PRO, Hemcon ChitoFlex^®^, OneStop™ Bandage and HemCon ChitoDot^®^, as well as skin substitute products that act as physical barriers (Chitosan Skin ^®^). Other products such as Beschitin^®^ W, Wellife^®^ LB-01, Tegasorb^®^ and TraumaStat^®^) comprise multifunctional dressings that promote skin protection and allows for tissue regeneration (Muzzarelli et al., 2016; Smith et al., 2016; Liu et al., 2018; Biranje et al., 2021).

Several studies have demonstrated the use of chitosan as a drug nanocarrier, with many hydrophilic or hydrophobic drugs encapsulated into chitosan nanocapsules, assessed by both *in vitro* and *in vivo* assays, used to treat different cancers. The most tested formulation to date comprises chitosan nanocapsules containing the antitumoral drug doxorubicin, a hydrophilic molecule. The monoclonal antibody transtuzumab has also been conjugated to DOX -chitosan nanoparticles to specifically target Her2+ cells in breast and ovarian carcinomas, displaying an increased uptake when compared to free DOX (Yousefpour et al., 2011). In another study, DOX was encapsulated in chitosan-pluronic micellae and 50 nm nanocapsules, presenting a high drug loading capacity and 77.33% higher therapeutic activity than free DOX against cultures MCF7 breast cancer cells (Naruphontjirakul and Viravaidya-Pasuwat, 2011). Finally, the use of hydrophobically modified chitosan nanoparticles in the delivery of silibinin, an antineoplastic flavo-lignan isolated from the seeds of the milk thistle plant (*Sylibum marianum*), promoted the solubility of this poorly water-soluble compound and the sustained release of the active compound (Kuen et al., 2017). In some cases, chitosan modifications are beneficial for the development of nanoantibiotic systems capable of antagonizing opportunistic pathogenic bacteria. Chitosan functionalized with 3,5-dinitro salicylic acid and linked to the antibiotic linezolid, for example, was shown to be effective against methicillin-resistant *Staphylococcus aureus* (MRSA), among other microorganisms (Teaima et al., 2020). In another assessment, a 2,3-dichloro-1,4-naphthoquinone grafted chitosan exhibited antimicrobial activities against *Staphylococcus aureus* and *Staphylococcus epidermidis*, presenting high effectiveness and low cytotoxicity, with high therapeutic indices, ensuring safe human tissue application (Pauli et al., 2023a).

In this context, the aim of this study was to formulate chitosan nanocapsules by the ionic gelation method employing tripolyphosphate polyanion as a crosslinker, loaded with 3-chloromethylene-menadione and 2,3-dichloro-1,4-naphthoquinone as the active pharmacological agent, to maintain tissues protected from bacterial infections and also able to regenerate into healthy fibroblastic tissue. Chitosan nanocapsules were characterized by the SEM, FTIR spectroscopy and DSC and TGA thermogravimetric techniques and the biological activities of the naphtoquinone-nanochitosan formulations were evaluated as antimicrobial drugs against bacterial species of clinical interest. The cytotoxicity of the formulations towards healthy surrounding tissue and their capability to improve cell migration in a wound healing assay were also investigated. Naphthoquinones nanoencapsulated into chitosan should be considered a potential adjuvant to topical medication against bacterial infections concomitantly associated to skin tissue regeneration.

## 2 Materials and methods

### 2.1 Experimental design

**FIGURE 1:**
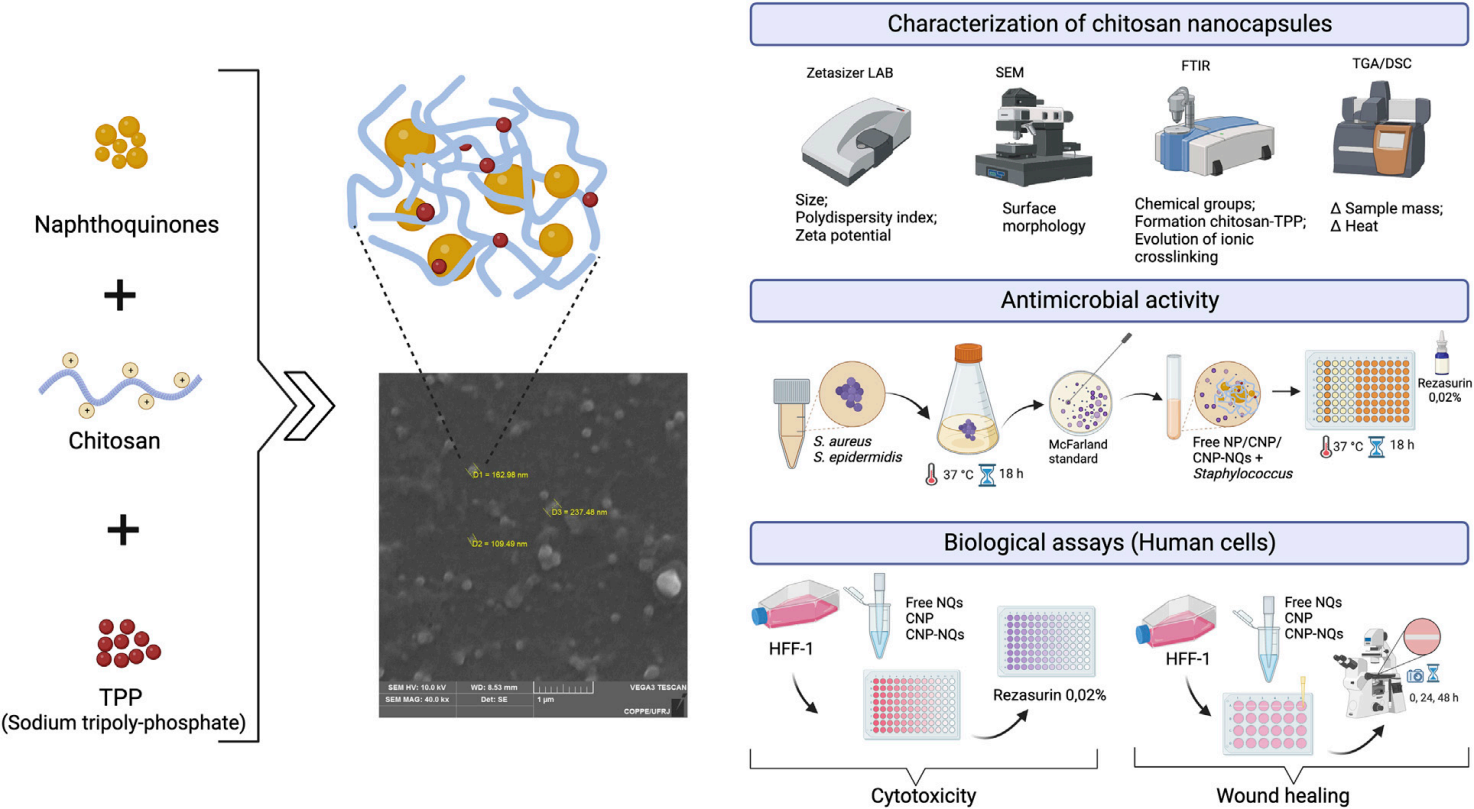
Experimental steps and analysis of effects of chitosan nanocapsules-loaded by naphthoquinones on human cells. The production of chitosan nanocapsules was carried out by the ionotropic gelatinization method and the resultant chitosan nanocapsules were characterized byphysicochemical and loaded by naphtoquinones. The citotoxicty of the formulations and evaluation of antimicrobial efficacy and wound healing were assyed in fibroblast cells.

The naphthoquinones 2-(chloromethyl)-3-methylnaphthalene-1,4-dione (IUPAC name) or 3-chloromethylene menadione (NQ1) and 2,3-dichloro-1,4-naphthoquinone (NQ2) were nanoencapsulated in chitosan using the ionotropic gelatinization method, as can be seen in Figure 1. After preparation of the nanocapsules containing naphthoquinones (CNP-NQs), physical-chemical characterization analyzes were carried out to investigate and confirm the nanoencapsulation, through SEM, FTIR, TGA and DSC. CNP-NQs underwent biological tests to assess their antimicrobial and wound healing efficacy, and cytotoxicity tests with healthy human cells, in order to assess their biocompatibility in preserving healthy tissues (Figure 1).

### 2.2 Synthesis of 2-(chloromethyl)-3-methylnaphthalene-1,4-dione (NQ2)

**FIGURE 2:**
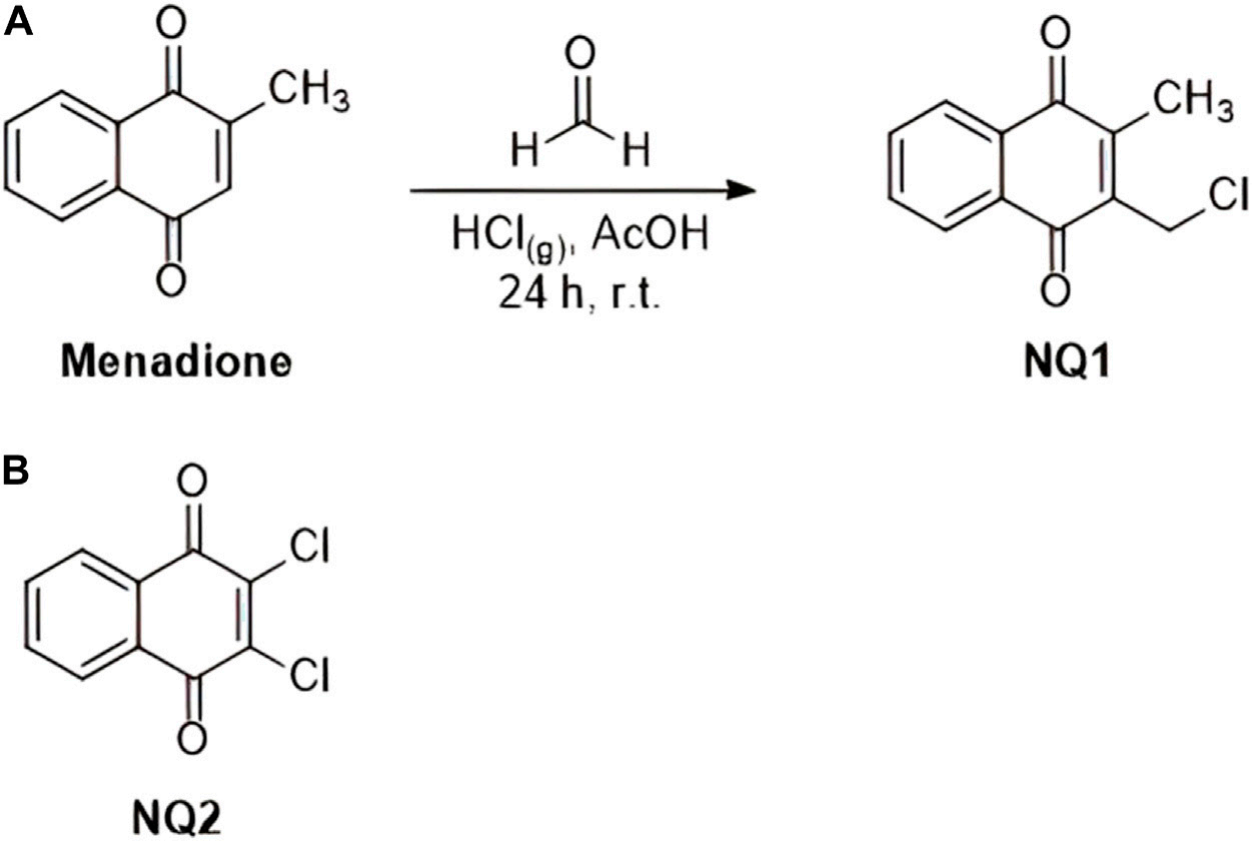
Chemical reaction applied for the synthesis of esize naphthoquinone 2-(chloromethyl)-3-methylnaphthalene-1,4-dione **(**NQ1) from commercial menadione **(A)**, as described above, and the chemical structure of the commercial naphthoquinone 2,3-dichloro-1,4-naphthoquinoneNQ2 **(B)**.

Menadione and naphthoquinone 2,3-dichloro-1,4-naphthoquinone (NQ2, Figure 2) were purchased from Sigma Aldrich Co MO, United States of America. Naphthoquinone 3-chloromethylene-menadione (NQ1) was synthetized from the commercial naphthoquinone as depicted in Figure 2, following a previously described method (Ribeiro et al., 2021). In brief, 5.8 mmol (1 g) of menadione, 300 mmol of acetic acid (16 mL) and 300 mmol of formalin (8 mL) were added to a 100 mL round bottom flask, kept on ice under stirring, followed by bubbling HCl gas into the solution for 20 min, and maintained at room temperature for 24 h. The mixture was then neutralized with sodium bicarbonate and extracted with ethyl acetate (3 × 50 mL) and the combined organic phases were dried with anhydrous sodium sulfate. Subsequently, the organic phase was evaporated and purified in a glass flash chromatographic silica gel column and eluted with a gradient mixture of hexane and ethyl acetate. A yellowish solid was obtained with a 95% yield, with the following characteristics:

**Melting point**: 100°C–101°C (p.f.lit.:107°C–108°C). **IR νmax (cm-1)**: 1292; 1332; 1378; 1440; 1458; 1592; 1621; 1663; 3043; 1H NMR (500.00 MHz, DMSO-d6): δ ppm 8.03–7.95 (m, 2H), 7.88–7.80 (m, 2H), 4.67 (s, 2H), 2.21 (s, 3H). 13C NMR; 125.00 MHz, DMSO-d6): δ ppm 184.74; 182.51; 146.84; 141.02; 134.64; 134.62; 132.07; 131.59; 126.51; 126.46; 36.82; 12.87.

### 2.3 Encapsulation of NQ1 and NQ2 naphthoquinones within nanochitosans

The chitosan solution was prepared using low molecular weight chitosan (50,000–190,000 Da) (Sigma-Aldrich Co) at 0.08%, solubilized in 1% acetic acid under constant stirring for 1 h at 45°C, after which the solution pH was adjusted to 4.6–4.8 with 5M sodium hydroxide (NaOH) and left under magnetic stirring for an additional 24 h at room temperature. The chitosan solution was then centrifuged at 12,429 x g for 30 min, filtered through a 47 mm pore membrane, and mixed with Tween 80 (0.1%) to avoid aggregates. The chitosan nanocapsules (CNP) were loaded with the NQ1 or NQ2 naphthoquinones at 2 mg/mL each mixed with 10 mL of the chitosan solution. Then, 5 mL of 0.08% sodium tripolyphosphate (TPP) (Sigma-Aldrich Co) were added to the empty chitosan or chitosannaphthoquinone mixture in a drop-wise manner under controlled and continuous dripping at 1 mL/min maintained under constant stirring for 40 min. Empty nanocapsules (CNP) fabrication took place as the same way described above without the addition of naphthoquinones NQ1 or NQ2. Subsequently, the solutions containing the CNP or CNP-NQs were centrifuged at 12,429 x g for 30 min, and the pellets were suspended in deionized water and ultrasonicated for 5 min using a SONIC ultrasonic probe (model 750 W) equipped with a 1/2 probe tip (constant duty cycle and 40% amplitude, 1725 J, 4°C, 5′) (Sonics and Materials Inc., CT, United States) (Gomes et al., 2016).

### 2.4 Efficiency of naphtoquinones encapsulation into nanochitosans

The encapsulation efficiency (EE) of naphtoquinones in the CNPs was determined by quantifying free naphthoquinones in the supernatants employing UV-Vis spectroscopy to estimate the entrapment of each compound, assessing Release profile of nanoencapsulated naphthoquinones, CNP-NQ1 and CNP-NQ2 (E), were kept at 36°C for 24 h. The release of naphthoquinones was quantified using UV-Vis spectroscopy to estimate the release of trapped compounds, evaluating absorbance at 335 nm for NQ1 and 344 nm for NQ2. at 335 nm for NQ1 and 344 nm for NQ2 on a Shimadzu MD spectrophotometer (United States). Encapsulation efficiencies were calculated by Equation 1 (Corrêa et al., 2019):

Equation 1. Encapsulation efficiencies of NQ1 or NQ2 (%)

$$Encapsulation\;efficiency\;\left[\%\right]=\frac{c_{\left(NQ\;added\right)}-c_{\left(NQ\;unencapsulated\right)}}{c_{\left(NQ\;added\right)}}\times 100$$

Where:

NQ added Is the initial concentration of NQ1 or NQ2 added to the reaction for encapsulation; NQ unencapsulated Is the concentration of NQ1 or NQ2 determined in the supernatants.

### 2.5 Kinetics release of naphthoquinones from chitosan nanocapsules

The release of naphthoquinones over time was evaluated using a UV-Vis spectrophotometer, at 335 nm for NQ1 and 344 nm for NQ2 on a Shimadzu spectrophotometer (MD, United States). Samples were incubated at 36°C under constant gentle agitation. At predetermined time intervals, 1 mL of the media was collected and NQ release was determined by a UV-Vis spectra analysis at every 1 h, 8 h and 24 h. After each measurement, the collected material was placed back into the system. Experiments were performed in triplicate to minimize error variations. Average values were used for subsequent data processing and plotting.

### 2.6 Chitosan nanocapsules size and stability determinations

Dynamic light scattering (DLS) was used to determine the average size and polydispersity indices (PdI) of the CNP and CNP-NQ formulations. Nanocapsules stability was determined from zeta potential (ZP) values, which are based on the electrophoretic mobility of the nanocapsules in aqueous suspensions. The samples were analyzed at a dispersion angle of 90° at 25°C, using a Zeta sizer LAB apparatus (Malvern Instruments, Malvern, United Kingdom).

### 2.7 Morphological characterization of nanocapsules

Scanning electron microscopy (SEM) was employed to study the surface morphology of the prepared chitosan nanocapsules. Samples drops were added to cover slips and dried at room temperature for 24 h. The dried samples were then mounted on stubs with conductive carbon tape and the surfaces sprayed without vacuum with an electrically conductive gold-palladium layer (20 nm thick). Images were visualized using a JEOL JSM-6460LV SEM (JEOL, CA, United States) taken by applying a beam accelerating voltage of 10 kV electrons.

### 2.8 Fourier transform infrared spectroscopy (FTIR) analysis

The FTIR spectra of the NQs, CNP and CNP-NQs were recorded on a Perkin Elmer 400 FTIR Spectrometer (Thermo Fisher, MA, United States) equipped with attenuated total reflectance (ATR). All spectra were recorded from 4000 to 600 cm^-1^ in the absorption mode at a 4 cm^-1^ resolution and 64 scans. The FTIR spectra were used to evaluate the chemical chitosan groups and investigate the formation of chitosan-TPP crosslink-networks. FTIR spectroscopy was also used to investigate the evolution of ionic crosslinks between naphthoquinones and CNP, comparing the FTIR spectra before and after NQ loadings.

### 2.9 Thermogravimetric and differential canning calorimetry analysis

Thermogravimetric curves (TGA) were obtained employing a TGA-60 Thermal Analyzer (Shimadzu) and differential scanning calorimetry (DSC) assessments were performed using a DSC-60 calorimeter (Shimadzu). Both analyses were carried out under a nitrogen atmosphere at a 50 mL/min flow rate and 10°C/min heating rate. Temperatures varied from 30°C to 500°C using about 5 mg of each solid sample.

### 2.10 Biological assays

#### 2.10.1 Microorganisms and human cell lineages

Five clinically relevant microorganisms, *Staphylococcus aureus* (ATCC 14458 and ATCC 29213), *Staphylococcus epidermidis* (ATCC 12228), *Streptococcus pyogenes* (ATCC 19615) and *Pseudomonas aeruginosa* (ATCC 15442), a pathogen resistant to various commercial germicides, used in the antimicrobial susceptibility tests were kindly provided by the FIOCRUZ-INCQS cell bank (RJ, BRA).

A healthy human fibroblast cell lineage, HFF-1 (ATCC SCRC-1041) was purchased from the Rio de Janeiro Cell Bank (BCRJ) for toxicological test assays following exposure to NQs, CNP and CNP-NQs performed *in vitro* through cell viability determinations and scratch assays.

#### 2.10.2 Evaluation of the antimicrobial activities of NQ1 and NQ2

Antimicrobial NQ1, NQ2, CNP, NQ1-CNP and NQ2-CNP activities were tested against *S. aureus*, *S. epidermidis*, *S. pyogenes* and *P. aeruginosa* by the microdilution method based on the Clinical and Laboratory Standards Institute (CLSI), with adaptations (Humphries et al., 2018). MIC (minimum inhibitory concentrations) were determined as the lowest sample concentration at which no microbial growth was observed. The 50% inhibitory concentrations (IC_50_) were predicted from inhibition curves constructed with increasing sample concentrations using resazurin dye as an indicator to identify viable cells.

*Staphylococcus aureus*, *Staphylococcus epidermidis*, *Streptococcus pyogenes* and *Pseudomonas aeruginosa* were inoculated in Mueller Hinton broth (MHB) containing 2.0 g/L of a meat extract, 17.5 g/L of casamino acids and 1.5 g/L of starch (KASVI, PR, BRA) and incubated at 37°C for 18 h under constant agitation and aerobic conditions. Subsequently, bacterial suspensions containing 10^8^ cells prepared according to the McFarland 0.5 scale were 10-fold serially diluted in MHB. Aliquots of free-NQs, CNP and CNP-NQs were 2-fold serially diluted, beginning at 5 mg/mL, and added to the bacterial suspensions at a final concentration of 10^7^ cells/mL in microplates and then incubated at 37°C for 18 h under constant agitation. Cell viability was assessed by adding 30 µL of 0.02% resazurin, followed by a further incubation at 37°C for 2 h according to McMillian, Li (McMillian et al., 2002). Fluorescence intensities were determined using a 2030 Multilabel Reader VICTOR™ X4 microplate reader (Perkin Elmer, MA, United States) at 530 nm (excitation) and 590 nm (emission).

The GraphPad Prism software, version 7 (GraphPad, CA, United States), was used to graphically represent inhibition percentages in relation to different sample concentration dilutions using a Log_10_ scale. Furthermore, inhibitory curves were generated with the purpose of predicting the IC_50_ for each tested sample dilution according to Li, Wang (Li et al., 2020) and Aragón, Villegas-Lelovsky (Aragón et al., 2023), with adaptations.

#### 2.10.3 Evaluation of the *in vitro* cytotoxicity of free and nano-encapsulated naphtoquinones and therapeutic indices calculations

Citotoxicity assays (CC_50_) concerning the free NQs, CNP and CNP-NQs were performed using healthy human HFF-1 fibroblast cells (ATCC SCRC-1041) on cell cultures at 5 × 10^5^ cells/mL plated in 96-well microplates in Dulbecco’s modified Eagle’s high glucose medium (DMEM, Ref# 11330–032) (Gibco, MT, United States) supplemented with 15% fetal bovine serum (FCS) (Corrêa et al., 2019). The microplates were incubated at 37°C for 24 h under a humidified atmosphere containing 5% CO^2^ for cell adhesion. The samples were 2-fold serially diluted, starting at 2.5 mg/mL and added to a semi-confluent cell monolayer followed by incubation for a further 24 h. Cell viability was assessed by adding 20 µL of 125 μg/mL resazurin to each well (Sigma-Aldrich Co.), according to McMillian, Li (McMillian et al., 2002) and fluorescence intensities were determined after 4 h of incubation using a Victor™ X microplate reader at 530 and 590 nm excitation and emission wavelengths, respectively.

The GraphPad Prism software, version 7 (GraphPad, CA, United States) was used to construct the growth inactivation plots for the human and bacteria cells for each tested sample in a log scale to determine the CC_50_ and IC_50_, respectively, for NQs, CNP and CNP-NQs. Therapeutic indices (TI) were calculated by evaluating the CC_50_/IC_50_ ratios of each sample.

#### 2.10.4 Migration ability of human healthy fibroblast cells treated with free or nano-encapsulated naphthoquinones–scratch assays

The cell migration assay was performed using the HFF-1 cell lineage cultured in 24-well plates (1.0 × 10^5^ cells/wells) in DMEM high glucose medium supplemented with 15% fetal bovine serum (FBS), at 37°C under a humidified atmosphere containing 5% CO_2_. After reaching confluence, a linear scratch was generated using a sterile plastic 200 µL-pipette tip of about 1.0 mm. Scattered cell fragments were removed by gentle washing with phosphate buffered saline (PBS). Non-damaged cells were then incubated with NQ1 (0.41 mg/mL), NQ2 (0.21 mg/mL), CNP-NQ1 (1.31 mg/mL), CNP-NQ2 (1.52 mg/mL) and CNP (equivalent to 1.5 mg/mL) formulations or only cells and the medium (negative control) maintained without FBS supplementation. The grooves were visualized in an inverted optical microscope (Biofocus, MG, BRA) at 0h, 24h and 48 h. Cell migration was evaluated by the cell-free area in relation to the initial area using the ImageJ program (public domain software offered by the National Institutes of Health - NIH, MD, United States), available at https://imagej.nih.gov/ij/download.html (Wu et al., 2020).

## 3 Results and discussion

### 3.1 Naphthoquinone-chitosan nanocapsules characterization: Size, morphology, Z potential, encapsulation efficiency and release kinetics

Herein, a simple and reliable method for the preparation of chitosan nanocapsules loaded with naphthoquinones was successfully employed, comprising a promising system for the delivery of poorly water-soluble drugs, such as the naphtoquinones, NQ1 and NQ2, in order to improve the bio-compatibility and solubility of these molecules.

The chitosan nanocapsules (CNP) were prepared by the ionotropic gelatinization technique, where the positively charged chitosan amine groups form electrostatic interactions with the negatively charged phosphate groups of polyanions such as TPP, leading to chitosan ionic gelling, resulting in the formation of spherical nanoparticles (De Campos et al., 2001; Qi et al., 2004; Zhao and Wu, 2006; Gierszewska and Ostrowska-Czubenko, 2016).

**FIGURE 3:**
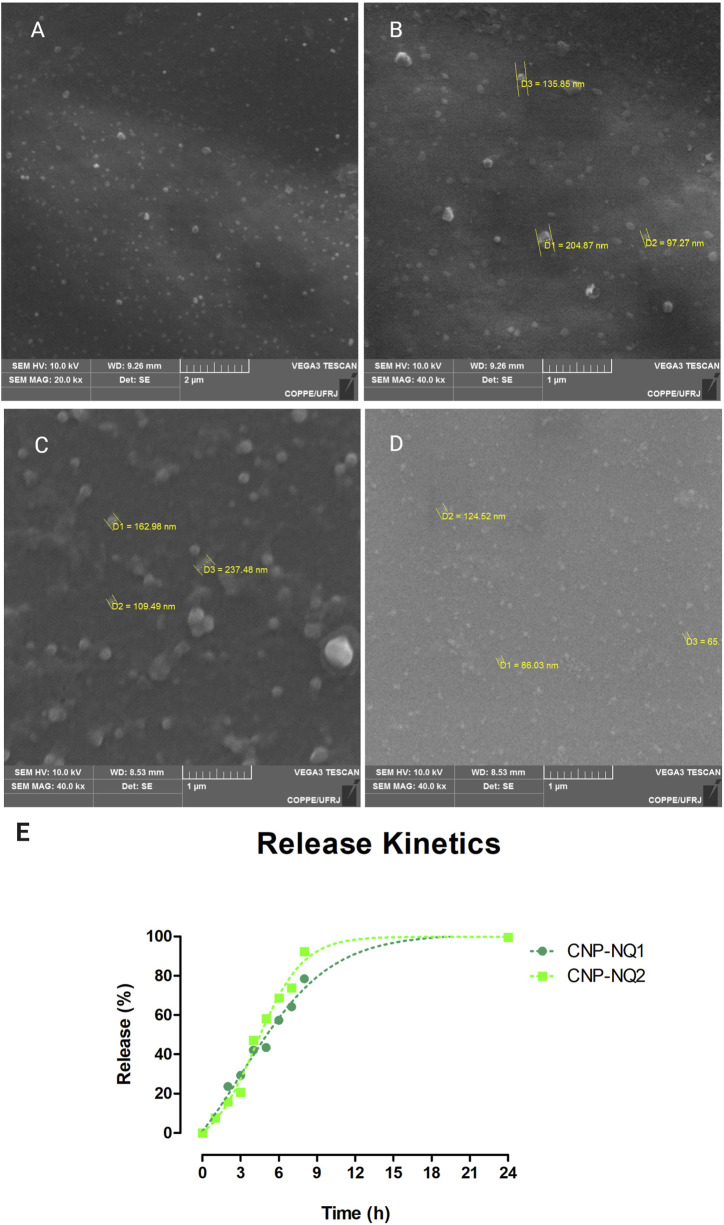
Morphological characterization of chitosan nanocapsules CNP **(A,B)**, CNP-NQ1 **(C)** and CNP-NQ2 **(D)**. A JEOL JSM-6460LV scanning electron microscope was used to visualize the nanocapsules and estimate their average sizes. Micrographs were taken at 10 kV and at ×20,000 (A and C) and ×40,000 **(B,D)** magnifications. Release profile of nanoencapsulated naphthoquinones, CNP-NQ1 and CNP-NQ2 **(E)**, were kept at 36°C for 24 h. The release of naphthoquinones was quantified using UV-Vis spectroscopy to estimate the release of trapped compounds, evaluating absorbance at 335 nm for NQ1 and 344 nm for NQ2.

Particle size measurements were carried out to characterize the obtained nanocapsules and evaluate their dispersion and aggregation characteristics, as these features can affect nanocapsule handling aiming at their application in biological systems. The hydrodynamic diameter distribution of the samples following a DLS analysis indicated a monomodal size distribution, with average sizes of 165 ± 2, 193 ± 2, 193 ± 9 nm for CNP, CNP-NQ1 and CNP-NQ2, respectively (Table 1). The morphological characteristics of the nanocapsules evaluated by SEM (Figure 3, panels A, B, C and D) indicated that CNP particles ranged from 97 nm to 205 nm CNP, CNP-NQ1 from 109 nm to 237 nm and CNP-NQ2 from 65 nm to 124 nm, following the size distribution ranges estimated by the DLS analysis (Table 1). The polydispersity indices (PdI) were established as 0.1 ± 0, 0.1 ± 0 and 0.1 ± 0 for CNP, CNP-NQ1 and CNP-NQ2, respectively (Table 1), very close to 0.1 in all samples, indicating homogeneous chitosan preparations (Abo Elsoud and El Kady, 2019).

**TABLE 1:** Size, polydispersity indices and encapsulation efficiency of the chitosan nano-capsules.

Samples	Size distribution (NM)	Average size (NM)	Polydispersity index (PDI)	ZP (MV)	Encapsulation efficiency (%)
CNP	69–361	165 ± 2	0.1 ± 0	+25	-
CNP-NQ1	80–420	193 ± 2	0.1 ± 0	+31	98
CNP-NQ2	64–568	193 ± 9	0.1 ± 0	+33	96

All experiments were performed in triplicate and the means and standard deviation were calculated (Taylor, 1997). Average sizes and polidispersity indices (PdI) were determined by dynamic light scattering (DLS) and Z potentials (ZP) was determined by electrophoretic light scattering (ELS). Encapsulation efficiency (EE) was calculated as described in Eq. 1 where the CNP-loaded naphtoquinones were determined by quantifying free naphthoquinone in the supernatants: EE (%) = C_(NQ, added)_—C_(NQ, unencapsulated)_/C_(NQ, added)_ X 100. NQ1—3-chloromethylene-menadione; NQ2—2,3-dichloro-1, 4-naphthoquinone; CNP, chitosan nanocapsule; CNP-NQ1, chitosan nanocapsules containing NQ1; CNP-NQ2, chitosan nanocapsules containing NQ2.

The Z potential for the CNP, CNP-NQ1 and CNP-NQ2 nanocapsules were determined as +25, +31 and +33 mV, respectively (Table 1), where positive ZP are explained by the presence of more positively charged chitosan molecules than negatively charged TPP (Chauhan et al., 2017). Zeta potentials of over 30 mV (absolute values) cause nanoparticles to repel each other in order to guarantee the physical colloidal suspension stability (Patravale et al., 2004). Therefore, the determined CNP-NQ1 and CNP-NQ2 values indicate colloidal stability and a low agglomeration trend. The initial pH of the chitosan solution emerges as a variable of significant relevance for nanoparticle formation, given that the charge densities of both chitosan and TPP are intrinsically dependent on this parameter. Chitosan solubility is increased by protonation of the -NH_2_ group when dissolved in acidic media. Considering that the p*K*_a_ of chitosan is around 6.5, most of its amino groups present positive charges within a pH range from 3.5 to 5.5. This indicates that chitosan would be positively charged because the media pH described herein ranges from 4.6–4.8. According to the p*K*_a_ of TPP (p*K*_a_ = 2.3), its charge density increases with increasing pH. Therefore, the inclusion of TPP, at higher pH, favors the formation of chitosan nanoparticles. Another factor that affects nanoparticle size and charge is the mass ratio between chitosan and TPP (chitosan/TPP ratio), where lower ratios culminate in decreased nanoparticle size. However, further decreasing the chitosan/TPP ratio leads to aggregation or the formation of larger nanoparticles (Ko et al., 2002; Karimi et al., 2013).

Previous studies have documented significant effects of the chitosan/TPP ratio on nanoparticle size. Variations in the size and Z Potential of nanoparticles have been demonstrated at different chitosan concentrations and pH variations. The Z Potential has been reported as decreasing with increasing pH, although particle size does not increase until the pH exceeds 5.5 (Antoniou et al., 2015). Other reports have concluded that if the pH ranges between 4.5 and 5, there is less −NH_3_^+^ neutralization during crosslinking, resulting in decreased particle size. When the initial pH exceeds 4.5, chitosan solution ionization is optimized for crosslinking with TPP. As the pH rises to above 5, approaching the chitosan p*K*_a_ of 6.5, lower protonation of amino groups takes place, leading to agglomeration and, therefore, the formation of larger particles (Gan et al., 2005; Hu et al., 2008). These findings are in agreement with the results obtained in the present study.

The EEs% for CNP-NQ1 and CNP-NQ2 were established as 98% and 96%, respectively, revealing good nanoencapsulation efficiencies for both naphthoquinones. The EE is an important parameter that informs nanocapsule loads, revealing the bioactive compounds amount loaded into nanoapsules as a percentage of the total concentration of the compounds added to the formulation. High encapsulation efficiencies are important, as they can reduce the amount of carriers required to deliver an efficient amount of active compounds to target sites, maintaining a minimal quantity in order to avoid citotoxicity by non-loaded carriers (Dos Santos et al., 2018).

To evaluate the performance of chitosan nanocapsules dispersed in aqueous media, release kinetics tests were conducted over 24 h. The chitosan nanocapsules loaded with NQ1 or NQ2 were able to release encapsulated naphthoquinones in a controlled manner. The time interval required for the release of 50% of the naphthoquinone loads was of about 5 h for CNP-NQ1 and 4 h for CNP-NQ2. About 80% of the naphthoquinone load from CNP-NQ1 was released in around 8 h, while 90% of the CNP-NQ2 load was released after 9 h (Figure 3E). These results indicate chitosan nanocapsules may comprise a remarkable strategy for the incorporation of active substances. Their noteworthy transport and controlled release capacity lies in their ability to enhance the absorption rates and bioavailability and ensure the targeted administration of drugs intended for different treatments. Nanochitosans can also optimize the solubilization and release of low water soluble drugs, as in the case of naphthoquinones, as well as promote the joint delivery of two or more drugs and protect therapeutic agents against undesirable degradation (Garg et al., 2019; Shaban et al., 2019).

### 3.2 Chitosan cross-link fourier transformed infrared spectroscopy (FTIR) analysis

**FIGURE 4:**
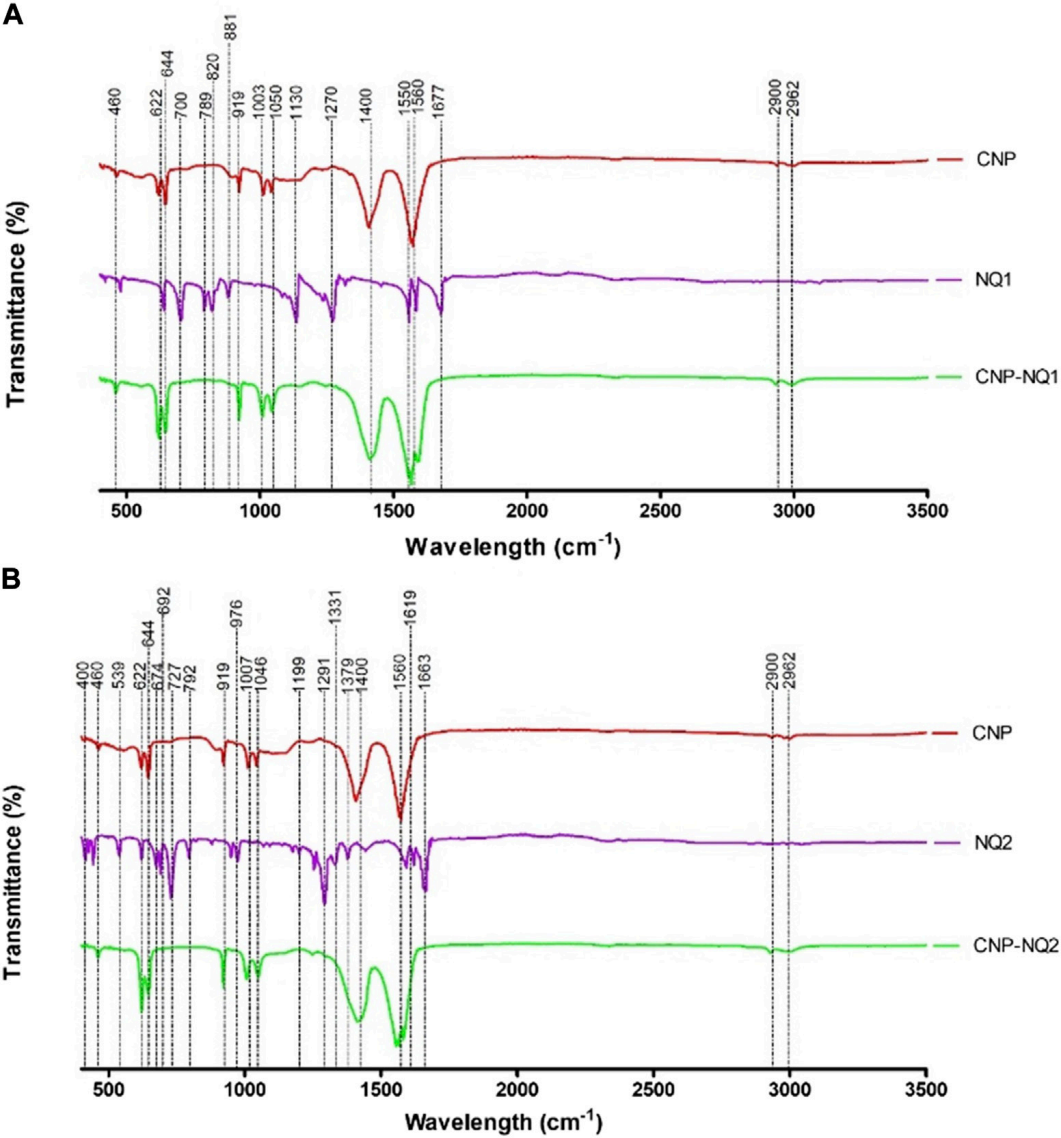
FTIR spectra of chitosan nanocapsules (CNP), 3-chloromethylenemenadione (NQ1) and chitosan nancoapsules loaded with NQ1 (CNP-NQ1) **(A)**; Chitosan nanocapsules (CNP), 2,3-dichloro-1,4-naphthoquinone (NQ2) and chitosan nancoapsules loaded with NQ2 (CNP-NQ2) **(B)**, were recorded on a Perkin Elmer 400 FTIR spectrometer equipped with ATR. All spectra were recorded from 4000 to 600 cm^−1^ in the absorption mode at a 4 cm^−1^ resolution and employing 64 scans. The spectra of each material (TPP, naphthoquinones and the chitosan solution) were acquired separately to compare the molecular patterns of each structure in each nanoformulation.

FTIR spectroscopy was employed to evaluate chemical chitosan groups and investigate the formation of cross-linked chitosan networks with TPP and the evolution of ionic crosslinking between chitosan and naphthoquinone, depicted in Figure 4.

The spectra relative to the empty nanocapsules (CNP) presented absorption bands characteristic of the chitosan matrix. Bands at 2962 and 2900 cm^-1^ correspond to the vibrational stretching of the C-H bonds from alkyl groups, while bands at 1500, 1400 and 1050 cm^-1^ are associated with the absorption of amino group bonds (NH_2_), vibration of hydroxyl groups (OH) and vibrations related to the C-O-C bonds present in the polymeric matrix, respectively (Hussein et al., 2018).

The infrared spectra of naphthoquinones NQ1 and NQ2 presented absorption bands characteristic of stretching of double carbon bonds and carbonyl oxygen bonds at 1677 cm^-1^ for NQ1, and at 1633 cm^-1^ for NQ2. Both NQs exhibited a prominent band from 1270 to 1290 cm^-1^, corresponding to C-O bond stretching. Furthermore, distinct bands were noted at 1500–400 cm^-1^, comprising naphthoquinone structural variation indicators, commonly referred to as the fingerprint region of these molecules. These absorption bands represent different bonds, including C-H, C-C, and C-O bonds (Singh et al., 2022).

Therefore, the absorption bands observed in the CNP and CNP-NQ spectra are quite similar, with no novel bands observed when the chitosan nanocapsules were loaded with NQ, corroborating previously described chitosan nanocapsule characteristics (Fernandes et al., 2011; Sotelo-Boyás et al., 2017; Xiao et al., 2017). Furthermore, the mass percentage (w/w) of the nanoencapsulated molecules in the final products, CNP-NQ1 and CNP-NQ2, are, 2.40% of NQ1 and 2.34% of NQ2, respectively, corroborating this result. These percentages support the prominent similarity of the FTIR spectra absorption bands of the empty nanocapsules (CNP) with the end products CNP-NQ1 and CNP-NQ2.

### 3.3 Thermogravimetric analysis

**FIGURE 5:**
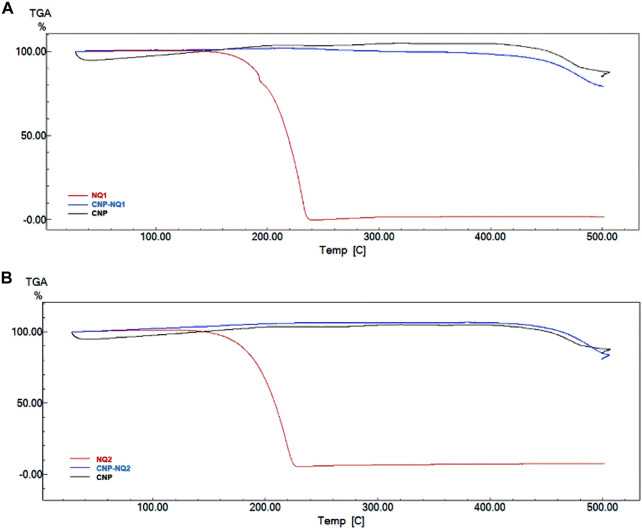
TGA thermograms. **(A)** NQ1, CNP and CNP-NQ1 samples; **(B)** NQ2, CNP and CNP-NQ2 samples. The thermogravimetric curves were obtained using a Shimadzu TGA-60 thermal analyzer under a nitrogen atmosphere with temperature varying from 30°C to 500°C, using about 5 mg of each solid sample. NQ1—3-chloromethylene-menadione, NQ2—2,3-dichloro-1,4-naphthoquinone, CNP—chitosan nanocapsules, CNP—chitosan nanocapsules, and NQ1 or NQ2-loaded chitosan nancoapsules (CNP-NQs).

A thermogravimetric curve analysis (Figure 5) indicated that the empty chitosan nanocapsules (CNP) have no mass loss regions as expected in commercial samples, which would be associated to residual water losses and polymer degradation (Maluin et al., 2019). These findings corroborate previously reports by Gadkari, Suwalka (Gadkari et al., 2019), where the zero mass loss noted for chitosan nanocapsules can be attributed to greater chitosan stability at the nanoscale when compared to commercial chitosan (Gadkari et al., 2019). All chitosan nanocapsules loaded with naphthoquinones (CNP-NQs) displayed similar thermo-decomposition to that noted for the empty nanocapsules, as CNP remains stable up to 500°C. Regarding the pure NQs, a 100% mass loss was noted from 150°C to 234°C, for both NQ1 and NQ2, referring to their respective decompositions (Hou et al., 2019). In addition, the absence of mass losses in regions associated to the degradation of loaded naphthoquinones in the CNP-NQ samples is indicative that nanocapsules are able to guarantee the thermal stability of the NQ-loaded material at the assay temperature, including biological system temperatures, preventing the decomposition of the NQ-encapsulated material.

### 3.4 Differential scanning calorimetry of NQ1-and NQ2-loaded nanocapsules

**FIGURE 6:**
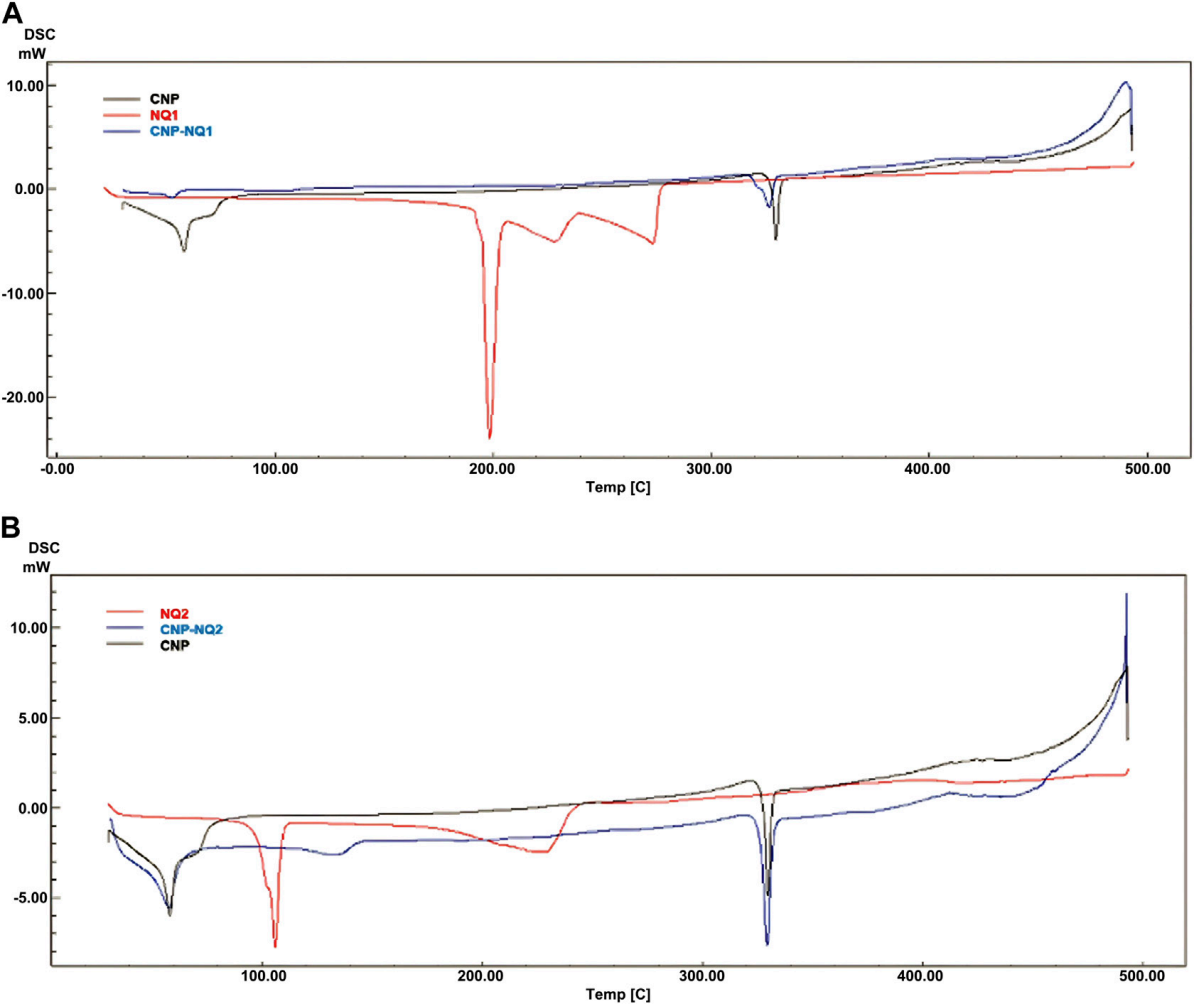
Calorimetric curves. **(A)** NQ1, CNP and CNP-NQ1; **(B)** NQ2, CNP and CNP-NQ2. Differential scanning calorimetry was performed using a Shimadzu DSC-60 calorimeter under a nitrogen atmosphere from 30°C to 500°C employing about 5 mg of each solid sample. NQ1—3-chloromethylene-menadione, NQ2—2,3-dichloro-1,4-naphthoquinone, CNP - chitosan nanocapsules, CNP—chitosan nanocapsules, and NQ1 or NQ2-loaded chitosan nancoapsules (CNP-NQs).

The DSC curves for the investigated NQs, CNP and CNP-NQ are depicted in Figure 6. The CNP thermograms indicated two endothermic events, the first at 33°C–82°C and the second, at 323°C–335°C, both previsouly described for free chitosan nanocapsules (Hosseinzadeh et al., 2012; Contri et al., 2014). The free naphtoquinones DSC curves demonstrated that NQ1 presents three consecutive endothermic events, the first from 188 °Cto 206°C, associated to naphthoquinone melting, and the second and third from 206°C to 276°C, probably associated with simultaneous sublimation and thermal decomposition events, with a 100% mass loss, previously noted in the TGA analysis (Figure 5A). NQ2, on the other hand, presents two successive endothermic events. The first takes place from 90°C to 111°C, reported by Sousa, da Silva (Sousa et al., 2012) as potentially associated to the melting range of naphthoquinones, and the second, from 136°C to 227°C, due to the thermal degradation of naphthoquinones, proven by a 100% mass loss observed in the TGA spectra at this temperature range (Figure 5), also in agreement with Sousa, da Silva (Sousa et al., 2012). The DSC curves of the naphthoquinone-loaded nanocapsules, CNP-NQ1 and CNP-NQ2, indicated no endothermic events similar to those observed regarding free naphthoquinones, indicating that the employed naphtoquinones were efficiently encapsulated by chitosan (Feyzioglu and Tornuk, 2016; Hadidi et al., 2020). In both cases, only endothermic events associated with CNP were observed, without any noticeable sample mass loss (Figure 6).

### 3.5 Antimicrobial effectiveness of free and nano-encapsulated naphthoquinones

**FIGURE 7:**
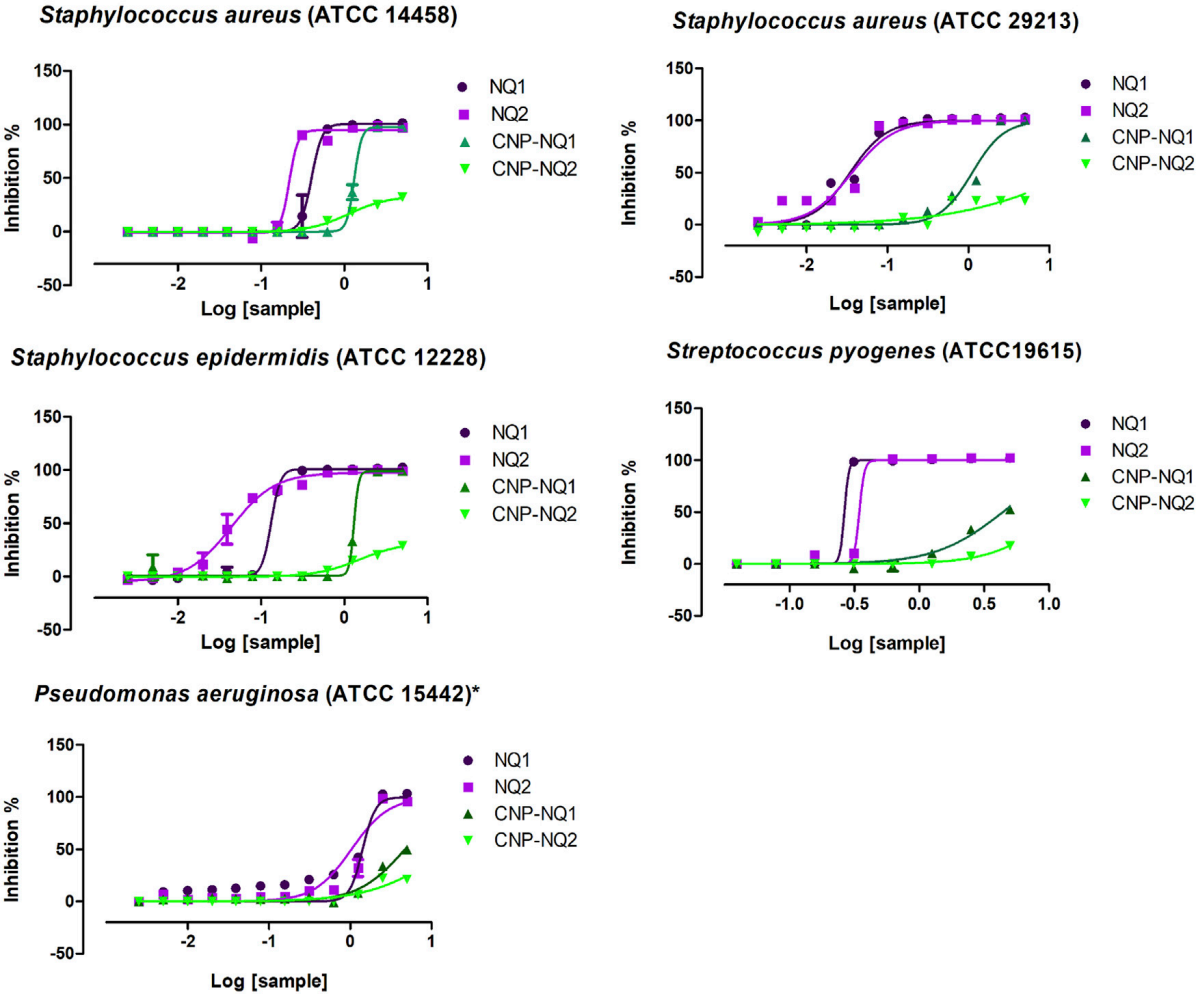
Bacteria inhibition curves. Dose-dependent responses of antimicrobial activities triggered by NQ1, NQ2, CNP-NQ1 and CNP-NQ2 at decreasing concentrations (5 mg/mL to 0.002 mg/mL) against *S. aureus* (ATCC 14458), *S. aureus* (ATCC 29213), *S. epidermidis*, *S. pyogenes* and *Pseudomonas aeruginosa*. Bacteria viability was monitored after 24 h exposure by the addition of resazurin, and curve fitting was performed using GraphPad Prism software, version 7 to estimate each IC_50_ shown in Table 2. All experiments were performed in triplicate. Sample concentrations are shown in *x*-axis as Log10. NQ1—3-chloromethylene-menadione, NQ2—2,3-dichloro-1,4-naphthoquinone, CNP—chitosan nanocapsules, CNP—chitosan nanocapsules, and NQ1 or NQ2-loaded chitosan nancoapsules (CNP-NQs).

The free naphthoquinones NQ1 and NQ2 were able to effectively inactivate *S. aureus* (ATCC 14458), *S. aureus* (ATCC 29213), *S. epidermidis* and *S. pyogenes* (ATCC 19615) growth, with low IC_50_ values varying from 0.03 to 0.4 mg/mL. On the other hand, *P. aeruginosa* were inhibited with IC_50_ values over 1 mg/mL (Table 2; Figure 7). *S. epidermidis* and *S. aureus* (ATCC 29213) seem to be more sensitive to both NQ1 and NQ2 than *S. aureus*, *S. pyogenes* and *P. aeruginosa* as the concentration required to inhibit both bacteria is lower when compared to the other assessed strains. NQ2 exhibited 2-fold and 3-fold higher antimicrobial activities compared to NQ1 against *S. aureus* and *S. epidermidis*. The antimicrobial efficiency of NQ2 against the other investigated bacteria did not reach 2-fold superiority compared to NQ1 (Table 2). Complete bacteria growth inhibition (MIC) was achieved by treatment with both NQ1 and NQ2, as depicted in Figure 7 and Table 2.

**TABLE 2:** Half-maximal inhibitory concentration (IC_50_) and minimum inhibitory concentration (MIC) of nano-encapsulated naphthoquinones against bacteria of clinical interest.

Sample	IC_50_ and MIC (mg/mL)									
	*Staphylococcus aureus* (ATCC 14458)		*Staphylococcus* epidermidis (ATCC 12228)		*Staphylococcus aureus* (ATCC 29213)		*Streptococcus* pyogenes (ATCC 19615)		*Pseudomonas aeruginosa* (ATCC 15442)*	
CNP	NI	NI	NI	NI	NI	NI	NI	NI	NI	NI
NQ1	0.4	1.2	0.1	0.3	0.03	0.2	0.3	0.3	1.04	2.5
NQ2	0.2	1.2	0.04	0.6	0.04	0.3	0.3	0.6	1.4	2.5
CNP-NQ1	1.3	2.5	1.3	2.5	1.1	2.5	4.4	>5.0	4.7	>5.0
CNP-NQ2	1.1	2.5	1.5	>5.0	>5.0	>5.0	>5.0	>5.0	>5.0	>5.0

NI, No inhibition up to 5 mg/mL. Antimicrobial activities evaluated as IC_50_ were estimated by the microdilution method using resazurin as the viability indicator to construct a concentration vs*.* proliferation inhibition curve. Experiments were performed in triplicate and IC_50_ values were estimated from inhibition curves using the GraphPad Prism v.8 software. The minimum inhibitory concentration (MIC) was determined as the lowest sample concentration at which no microbial growth was observed. NQ1—3-chloromethylene-menadione, NQ2—2,3-dichloro-1, 4-naphthoquinone; CNP, chitosan nanocapsules; CNP, chitosan nanocapsules, and NQ1 or NQ2-loaded chitosan nancoapsules (CNP-NQs). *Asterisk indicates that *P. aeruginosa* is a resistant pathogen to various commercial germicides.

Following chitosan nanoparticle encapsulation, both CNP-NQ1 and CNP-NQ2 formulations required higher concentrations to promote the same antimicrobial effect of their free counterparts, as their IC_50_ increased to 1.3 mg/mL and 1.1 or 1.5 mg/mL to inhibit *S. aureus* (ATCC 14458) and *S. epidermidis,* respectively (Table 2; Figure 7). The inhibition of *S. aureus* (ATCC 29213), *S. pyogenes* and *P. aeruginosa* by CNP-NQ1 was achieved with IC_50_ = 1.1 mg/mL, 4.4 mg/mL and 4.7 mg/mL, respectively, with superior efficiency inhibition curves compared to CNP-NQ2. Complete inhibition (MIC) upon CNP-NQ1 treatment was achieved against *S. aureus* (ATCC 14458), *S. epidermidis* and *S. aureus* (ATCC 29213), but not against *S. pyogenes* and *P. aeruginosa*. The inhibition curves produced by CNP-NQ2 treatment did not achieve 50% of antimicrobial activity against *S. aureus* (ATCC 29213) *S. pyogenes* and *P. aeruginosa* and, hence, their IC_50_ were recorded as >5 mg/mL, the highest tested concentration. Complete growth inhibition (MIC) using CNP-NQ2 was only achieved against *S. aureus* (ATCC 14458) (Table 2; Figure 7).

Although chitosan is frequently reported as harboring intrinsic antibacterial ability, the non-loaded chitosan nanoparticles investigated herein were unable to inactivate bacteria growth up to 5 mg/mL, indicating no nanopolymer contribution to the antimicrobial ability of the developed CNP-NQs (Table 2). Various intrinsic and extrinsic factors can influence antimicrobial chitosan activity, including polymer deacetylation degree, environmental pH or ionic strength, polymer molecular weight, chelating capacity and, finally, the physical state of the applied chitosan (Yilmaz Atay et al., 2019).

Both *S*. *aureus* and *S. epidermidis* can represent human health risks. *Staphylococcus epidermidis* is part of the human skin microbiota and participates in the maintenance of skin homeostasis, preventing the colonization of the dermal tissue by pathogenic bacteria, such as *S. aureus*. Although *S. epidermidis* exhibit low virulence, this bacterium is most frequently associated to nosocomial infections, becoming more worrisome than *S. aureus*, although the latter is considered a highly virulent pathogen (Brown and Horswill, 2020; Roy et al., 2020; Severn and Horswill, 2023a). An imbalance in the abundance of *S. epidermidis* populations, particularly in immunosuppressed individuals, may also result in unhealthy skin conditions such as atopic dermatitis, rosacea, seborrheic dermatitis, dandruff and impaired post-surgical wound healing (Severn and Horswill, 2023b). *Staphylococcus aureus* is among the main morbidity and mortality causes following colonization by infectious agents, and is also frequently associated to nosocomial conditions and severe skin infections (Cheung et al., 2021). *Streptococcus pyogenes* is an exclusive contagious human pathogen that infects individuals through contact via the oral cavity, skin and wounds, mainly causing moderate skin and oropharynx infections, but can also be associated with severe and invasive infections depending on the strain (Fiedler et al., 2015; Rohde and Cleary, 2022). *Pseudomonas aeruginosa* is an opportunistic life-threatening pathogen, leading to nosocomial infections that can be fatal to immunocompromised individuals. Wounds infected with *P. aeruginosa* can become a serious problem due to their ability to form biofilms, which confer resistance against treatment and superior colonization capacity associated with long-term persistence, impairing complete healing (Thi et al., 2020).

Therefore, avoiding the infection or controlling the proliferation of such bacteria through nano-encapsulated NQ1 or NQ2 would be an interesting strategy to promote prolonged and controlled release of antimicrobial agents with preserved potential in nano-encapsulated formulations, promoting skin health maintenance.

### 3.6 Curative potential of nano-encapsulated naphthoquinones estimated through wound healing ability

**FIGURE 8:**
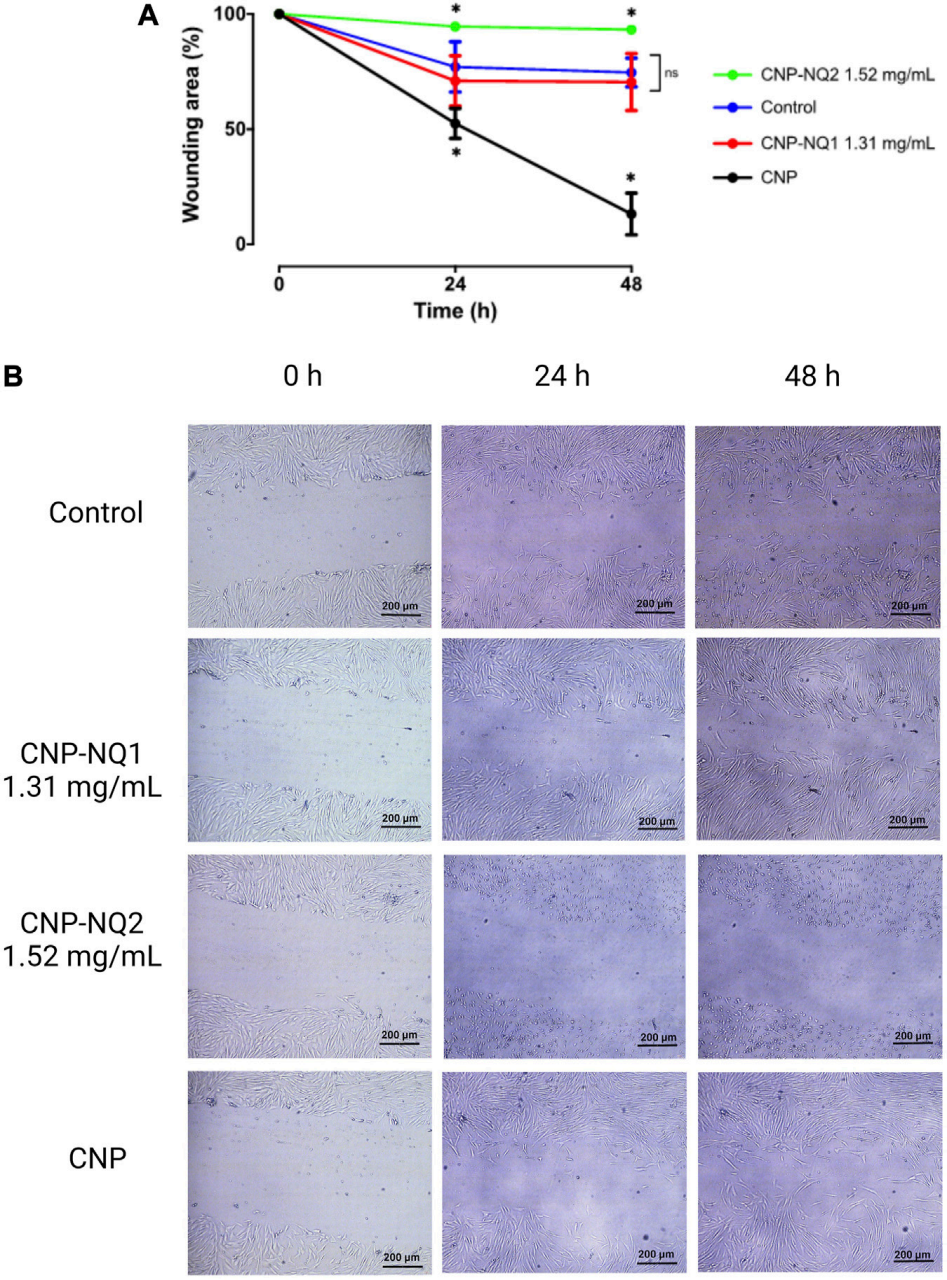
Kinetics of HFF-1 human fibroblast migration following challenges with CNP, CNP-NQ1 and CNP-NQ2 for 24 h and 48 h **(A)**. Representative photomicroscopies of the wound areas at 0 h, 24 h and 4 8 h after challenges with CNP, CNP-NQ1 and CNP-NQ2 **(B)**. All experiments were performed in triplicate and statistical significances were evaluated by a two-way ANOVA followed by a post-test Tukey test considering *p < 0.05 as a significant difference compared to control. (ns) non-significant. NQ1—3-chloromethylene-menadione, NQ2—2,3-dichloro-1,4-naphthoquinone, CNP—chitosan nanocapsules, CNP - chitosan nanocapsules, and NQ1 or NQ2-loaded chitosan nancoapsules (CNP-NQs).

The curative potentials of the CNP-NQ1 and CNP-NQ2 on dermal tissues were estimated through the effects of the nano-encapsulated naphthoquinones on wound healing. Monolayer cells from the human HFF-1 fibroblast line were scratched, leaving a cell-free open area, and cells were challenged with CNP-NQ1 and CNP-NQ2 at 1.3 mg/mL and 1.5 mg/mL, respectively. The open area was monitored for 24 h and 48 h and the migration pattern of HFF-1 cells documented by photomicroscopies (Figure 8). As free naphthoquinones NQ1 and NQ2 exhibit a poor solubility in water their ability to stimulate healthy cell migration compared to the nano-encapsulated compounds could not to monitored (Supplementary Figure S1).

Chitosan nanocapsules (CNP) were able to stimulate fibroblast migration, leaving a wound area of about 10%, significantly higher than the control group, where fibroblasts cells were able to reach a wound area of about 75% in 48 h. CNP-NQ1 stimulated fibroblast migration similarly to the control culture, while no wound area closure was observed after treatment with CNP-NQ2 (Figure 8A). Fibroblast cells treated with CNP and CNP-NQ1 exhibited a healthy and highly refringent morphology, with a typical fibroblastic shape and completely adhered to the bottom plate, indicating no toxicity following 48 h of treatment (Figure 8B and Supplementary Figure S1).

On the other hand, the photomicroscopys’s analyses obtained from HFF-1 cultures challenged with CNP-NQ2 suggests an important citotoxicity of this formulation since the fibroblast cells exhibited an unhealthy appearance with round shape and starting to lose the adherence, which justifies the absence of migratory cells in the wounding area (Figure 8B; Supplementary Figure S1).

Wound healing is a multistage process during when the damaged tissue is reconstituted physically and functionally. A variety of medicine formulations or devices can be employed to accelerate the wound healing process and prevent opportunistic infections. Among these compounds, chitosan and its several derivatives, such as hydrogel, sponge, film, scaffold, nanoparticle, sulfate or sulfonated-chitosan, among others, can have a positive effect on wound healing. Chitosan is able to interfere in the first three stages; hemostasis, inflammation, proliferation and migration, accelerating wound closure as demonstrated by the migration assay (Matica et al., 2019; Feng et al., 2021; Ferreira et al., 2022; Pauli et al., 2023b; Ferreira et al., 2023) (Figure 8, panels A–B). It has been reported that chitosan promotes blood clot formation by stimulating the expression of the adhesion molecule GP IIb-IIIa at the surface of activated platelets and by stimulating platelet aggregation via electrostatic interactions between positively charged chitosan and negatively charged molecules at the surface of activated platelets. Chitosan can also electrostatically interact with erythrocytes and their negatively charged neuraminic acid residues to become part of the blood clot. A successful hemostatic effect is completed by chitosan-induced inhibition of plasmin release that prevents clot dissolution. During the inflammation stage, chitosan can assist by avoiding or combating bacterial infection, as its positive charge allows it to bind with negatively charged carbohydrate, lipid and protein residues located on the cell surface of bacteria, consequently inhibiting their growth (Kim, 2018; Khanna et al., 2020). Furthermore, chitosan exerts anti-inflammatory effects that reduce inflammatory cytokines, IL-10 and TNF-α, reducing oxidative damage and inflammation (Yang et al., 2016; Mohyuddin et al., 2021). However, as mentioned above, antimicrobial effectiveness depends on a variety of intrinsic and extrinsic factors (Matica et al., 2019; Feng et al., 2021; Ferreira et al., 2022; Pauli et al., 2023b; Ferreira et al., 2023). Moreover, chitosan can stimulate the proliferation of fibroblasts, which compose the remodeled tissue during wound healing. A previous report (Howling et al., 2001) demonstrated that cultures of human fibroblasts treated with chitosan result in a proliferative response dependent on the presence of fetal bovine serum. It is postulated that chitosan acts indirectly by complexing with serum components, such as heparin, cytokines, and growth factors, potentiating their mitogenic activity, which could explain the apparent proliferative effect of CNP on cultured fibroblasts compared to the control group, as depicted in Figure 8B.

### 3.7 Viability of healthy skin cells challenged with the nano-encapsulated naphthoquinones–therapeutic index determinations

To study the margin of safety and efficacy of the novel nano-encapsulated naphtoquinones investigated herein, cultures of healthy human HFF-1 fibroblasts were challenged with CNP-NQ1 and CNP-NQ2 for 24 h. Their 50% cytotoxic concentration (CC_50_) and the therapeutic index (TI), comprising the ratio between CC_50_ and IC_50,_ were calculated (Table 3), expressing how distant the toxic concentration is from the effective pharmacological agent concentration (Muller and Milton, 2012; Indrayanto et al., 2021).

**TABLE 3:** Half-maximal cytotoxic concentrations (CC_50_) of nanoencapsulated naphthoquinones and their antimicrobial therapeutic indices.

Estimated indices	Cell lineage or bacterial strain	Formulations			
		NQ1	NQ2	CNP-NQ1	CNP-NQ2
CC_50_ (mg/mL)	HFF-1 (HTB-26)	0.005	0.03	132	1.1
TI	*Staphylococcus aureus*	0.01	0.1	104*	0.95
	ATCC 14458				
	*Staphylococcus epidermidis* ATCC 12228	0.04	0.2	100*	0.70
	*Staphylococcus aureus*	0.16	0.83	120*	-
	ATCC 29213				
	*Streptococcus* pyogenes	0.02	0.08	30*	-
	ATCC 19615				
	*Pseudomonas aeruginosa*	0.005	0.02	28*	-
	ATCC 15442**				

TI, Therapeutic index calculated as the CC_50_/IC_50_ ratio, where one asterisk (*) indicates TI ≥ 100 and > 27, 10-fold or 2.7-fold higher than the TI, value considered as safe, indicating that the antimicrobial agent may be safely used in human tissues. NQ1—3-chloromethylene-menadione, NQ2—2,3-dichloro-1, 4-naphthoquinone; CNP, chitosan nanocapsules, CNP-NQ1—NQ1-loaded chitosan nanocapsules (CNP-NQs). CNP-NQ2—NQ2-loaded chitosan nanocapsules. **Asterisk indicates that *P. aeruginosa* is a resistant pathogen to various commercial germicides.

Unsurprisingly, NQ1 and NQ2 exhibited low therapeutic indices, ranging from 0.005 to 0.8, against all investigated bacterial species, indicating that their toxic concentration is higher than their effective concentration and, thus, cannot be safely administered in their free form (Table 3). The safety *status* of NQ1 was improved upon chitosan nanoparticle encapsulation, indicated by an increased CNP-NQ1 CC_50_ value of 132 mg/mL which, when compared to its IC_50_, resulted in therapeutic indices of 104, 100 and 120 for *S. aureus* **(**ATCC 14458) and *S. epidermidis* and *S. aureus* (ATCC 29213), respectively. Therapeutic indices for *S. pyogenes* and *P. aeruginosa* were 29.90 and 27.94, respectively, which are not as efficient as those mentioned previously, but still considered safe for application (Table 3).

An adequate therapeutic index is considered as ≥ 10, meaning that the effective concentration should be at least 10-fold higher than its cytotoxic concentration, ensuring safe use of a certain compound without the threat of side effects (Muller and Milton, 2012; Indrayanto et al., 2021). Quinone-containing compounds are known to be harmful to biological systems by nonspecifically reacting to several compounds such as electron transfer agents or electrophiles, thus able to promote beneficial health effects while also generally accompanied by high toxicity (Kumagai et al., 2012). The low TI of both free naphthoquinones investigated herein can be overcome by their encapsulation in chitosan. However, although the CC_50_ value calculated for CNP-NQ2 increased 35-fold when compared to NQ2, its therapeutic index is < 1 or could not be determined, indicating that this compound’s effective antimicrobial concentration is too close to or above its CC_50_, impairing its safe application to treat human infections, even if topically (Table 3).

Bacterial wound infection can impair the healing process from the inflammation phase to the proliferation and remodeling phases, leading to healing failure. In this regard, wound treatment by effective antimicrobial agents is critical to ensure complete dermal tissue regeneration (Öhnstedt et al., 2019). Considering that chitosan, the encapsulating material, harbors intrinsic wound healing properties with no toxicity and desirable biocompatibility, the encapsulation of NQ1 into nano-chitosan, a recognized antimicrobial agent able to inactivate *S. aureus*, *S. epidermidis, S. pyogenes* and *P. aeruginosa* should be considered a promising wound care formulation.

## 4 Conclusion

An efficient naphthoquinone encapsulation method was developed herein, improving naphthoquinone solubility, and reducing toxicity, thus enabling their safe application, particularly nano-encapsulated NQ1 in chitosan. The nano-encapsulation process retained naphthoquinone antimicrobial effects with desirable therapeutic indices, and the ability to promote wound care, *i.e.,* highlighting the curative potential of CNP-NQ1 to protect injured skin tissue from bacterial contamination, preventing skin infections. Furthermore, CNP-NQ1 may contribute to accelerate the healing process, ultimately leading to complete dermal tissue recovery. By harnessing the biocompatible and biodegradable properties of chitosan, this study presents a promising approach for the development of pharmaceutical formulations displaying enhanced antimicrobial properties and wound healing capabilities.

## Data Availability

The original contributions presented in the study are included in the article/Supplementary Material, further inquiries can be directed to the corresponding author.
