# Supplementary material for: Antimicrobial and wound healing potential of naphthoquinones encapsulated in nanochitosan
Source: Front Bioeng Biotechnol. 2024 Jan 4;11:1284630. doi: 10.3389/fbioe.2023.1284630 (PMC10794614; doi:10.3389/fbioe.2023.1284630)
Supplement: Supplementary file 1 [file DataSheet1.DOCX]

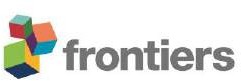


*Supplementary Material*

**Supplementary Figures**


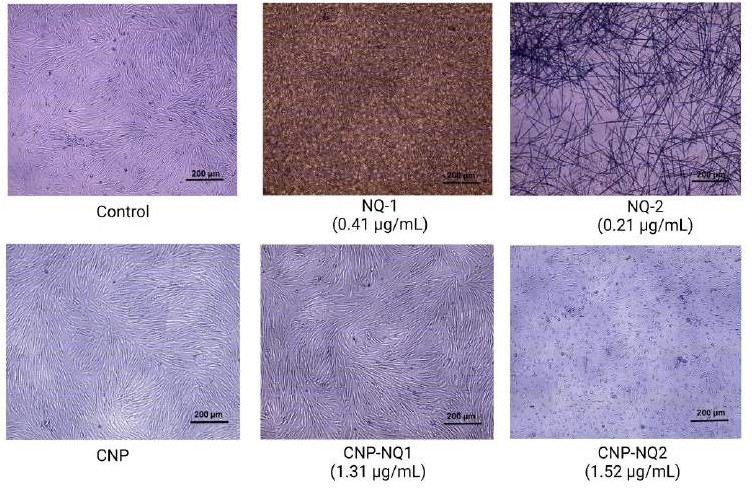


**Supplementary Figure 1** Representative photomicroscopies of HFF-1 cells challenged with CNP, NQ1, NQ2, CNP-NQ1 and CNP-NQ2 for 48h obtained during wound healing assays, depicting a general cell morphology view. NQ1 - 3-chloromethylene- menadione; NQ2 - 2,3-dichloro-1,4-naphthoquinone; CNP – chitosan nanocapsules; CNP-NQ1 - chitosan nanocapsules containing NQ1; CNP-NQ2 - chitosan nanocapsules containing NQ2.
